# Epidemiology and Heritability of Major Depressive Disorder, Stratified by Age of Onset, Sex, and Illness Course in Generation Scotland: Scottish Family Health Study (GS:SFHS)

**DOI:** 10.1371/journal.pone.0142197

**Published:** 2015-11-16

**Authors:** Ana Maria Fernandez-Pujals, Mark James Adams, Pippa Thomson, Andrew G. McKechanie, Douglas H. R. Blackwood, Blair H. Smith, Anna F. Dominiczak, Andrew D. Morris, Keith Matthews, Archie Campbell, Pamela Linksted, Chris S. Haley, Ian J. Deary, David J. Porteous, Donald J. MacIntyre, Andrew M. McIntosh

**Affiliations:** 1 Division of Psychiatry, University of Edinburgh, Edinburgh, United Kingdom; 2 Institute of Genetics and Molecular Medicine, University of Edinburgh, Edinburgh, United Kingdom; 3 The Patrick Wild Centre, University of Edinburgh, Edinburgh, United Kingdom; 4 Division of Population Health Science, University of Dundee, Dundee, United Kingdom; 5 College of Medical, Veterinary and Life Sciences, University of Glasgow, Glasgow, United Kingdom; 6 Usher Institute of Population Health Sciences and Informatics University of Edinburgh, Bioquarter, Edinburgh, United Kingdom; 7 Division of Neuroscience, University of Dundee, Dundee, United Kingdom; 8 Generation Scotland, Centre for Genomic and Experimental Medicine, Institute of Genetics and Molecular Medicine, University of Edinburgh, Edinburgh, United Kingdom; 9 MRC Human Genetics Unit, Institute of Genetics and Molecular Medicine, University of Edinburgh, Edinburgh, United Kingdom; 10 Centre for Cognitive Ageing and Cognitive Epidemiology, Department of Psychology, University of Edinburgh, Edinburgh, United Kingdom; University of Oxford, UNITED KINGDOM

## Abstract

The heritability of Major Depressive Disorder (MDD) has been estimated at 37% based largely on twin studies that rely on contested assumptions. More recently, the heritability of MDD has been estimated on large populations from registries such as the Swedish, Finnish, and Chinese cohorts. Family-based designs utilise a number of different relationships and provide an alternative means of estimating heritability. Generation Scotland: Scottish Family Health Study (GS:SFHS) is a large (n = 20,198), family-based population study designed to identify the genetic determinants of common diseases, including Major Depressive Disorder. Two thousand seven hundred and six individuals were SCID diagnosed with MDD, 13.5% of the cohort, from which we inferred a population prevalence of 12.2% (95% credible interval: 11.4% to 13.1%). Increased risk of MDD was associated with being female, unemployed due to a disability, current smokers, former drinkers, and living in areas of greater social deprivation. The heritability of MDD in GS:SFHS was between 28% and 44%, estimated from a pedigree model. The genetic correlation of MDD between sexes, age of onset, and illness course were examined and showed strong genetic correlations. The genetic correlation between males and females with MDD was 0.75 (0.43 to 0.99); between earlier (≤ age 40) and later (> age 40) onset was 0.85 (0.66 to 0.98); and between single and recurrent episodic illness course was 0.87 (0.72 to 0.98). We found that the heritability of recurrent MDD illness course was significantly greater than the heritability of single MDD illness course. The study confirms a moderate genetic contribution to depression, with a small contribution of the common family environment (variance proportion = 0.07, CI: 0.01 to 0.15), and supports the relationship of MDD with previously identified risk factors. This study did not find robust support for genetic differences in MDD due to sex, age of onset, or illness course. However, we found an intriguing difference in heritability between recurrent and single MDD illness course. These findings establish GS:SFHS as a valuable cohort for the genetic investigation of MDD.

## Introduction

Major depressive disorder (MDD) is a highly prevalent psychiatric disorder that is now the leading cause of worldwide disability in terms of years lived with disability [1]. In the majority of Western countries, the lifetime prevalence of MDD typically varies between 8% and 12% [2, 3]. There are consistently established relationships with female gender, alcohol misuse, and marital dissatisfaction or divorce [3–7]. The high prevalence and disability associated with MDD make research aimed at understanding its aetiology and developing effective treatments a priority.

MDD aggregates within families and the heritability of MDD has been estimated as 37% (SE 5%) in a meta-analysis of twin studies [8] and 32% (SE 9%) using genomic similarity among unrelated individuals [9]. Given the genetic contribution to MDD, genetic studies are a potential means of understanding its aetiology as well as identifying new drug targets. Despite this substantial genetic contribution to its aetiology, candidate gene [10] and genome-wide association studies [11], including a mega-analysis of more than 20,000 individuals with 9240 cases and 9519 controls in the discovery sample [12], have failed to identify significantly associated specific genetic variants [13]. Nonetheless, genome-wide association and related studies have shown that MDD is a genetically complex disorder [14] where risk is proposed to result from the cumulative effects of many low-penetrance genetic variants [9, 12].

Increasingly it is also recognised that a diagnosis of MDD may group together individuals who suffer from causally distinct conditions. Some studies indicate that the heritability estimates of MDD differ by sex [15, 16] with female MDD showing higher heritability than male MDD [16] suggesting that the genetic causes may be somewhat distinct [15]. Further, it has been suggested that both age of onset and single versus recurrent episode illness course may have somewhat differing genetic aetiologies [17, 18]. These findings highlight the substantial heterogeneity of MDD, which may further impede the search for genetic causes [19].

There is therefore an urgent need to increase the sample sizes available for GWAS and to refine and stratify the phenotype to identify subtypes of MDD that are more genetically homogenous, and better targets for association studies. Pedigree-based genetic studies are an efficient means for dissecting trait heterogeneity because they are able to capture all additive heritability whilst matching for key confounds present in studies of unaffected subjects [20]. The ability to study the co-segregation of MDD and genetic variants within families has the potential to identify highly homogeneous subsets of individuals with less complex genetic architectures for MDD and more readily identifiable and penetrant risk factors. These rare forms of MDD may then inform further studies of common genetic risk factors–much as they have done in the study of Alzheimer’s disease [21].

Generation Scotland: the Scottish Family Health Study (GS:SFHS) is a large (n = 20,198) population-based family study with high-fidelity phenotyping for MDD [22]. Volunteer participants were identified from the general population and assessed for a lifetime prevalence of MDD using structured diagnostic interviews. In the current study we seek to estimate the prevalence and heritability of MDD in this large Scottish sample. In order to benchmark GS:SFHS against other cohorts, known associations with established sociodemographic risk factors were identified and their effect sizes estimated. Finally, we sought to identify more homogeneous subgroups of MDD by stratifying affected subjects by gender, age of onset, and clinical course. The heritability of these subgroups and the genetic correlations for MDD between them was tested as a means of estimating their utility for linkage and association studies. The genetic correlation between subgroups of MDD was also evaluated as a means of identifying whether the stratifications yielded more genetically distinct targets for further investigation.

## Materials and Methods

### Participants

GS:SFHS is a population-based sample designed to identify the genetic causes of common complex diseases. The complete study protocol and other summary characteristics have been described in detail elsewhere [22, 23]. The participants were recruited from primary care general medical practitioner registries (GPs) across Scotland blindly to health status. Identification of individuals through GP registries should not bias population recruitment because, in the UK, approximately 96% of the population is registered with a GP [24]. Many conditions were assessed, including MDD and other common conditions such as cardiovascular illness, hypertension and chronic pain. In order to minimise ascertainment bias, MDD-affected subjects were neither actively recruited nor used to recruit related MDD-affected participants. All participants were asked to refer at least one relative to the study, but neither recruitment nor referral of a relative was dependent on the diagnostic status of any particular condition or health outcome. Participants were informed the purpose of the study was to study the health of the Scottish population.

Recruitment from GP practices was initially limited to 35–65 year olds (2006–2010), but the age criterion was later relaxed (2010) to include relatives from the ages of 18 years and older. Individuals were invited to participate and to identify at least one first-degree relative, aged 18 or over, who would participate. Recruitment was also initially limited to GP practices in Glasgow and Tayside and subsequently extended in 2010 to include Ayrshire, Arran and Northeast Scotland. Relatives of recruited individuals could come from any location. Data collection took place between February 2006 and March 2011. Around 126,000 random individuals who were identified from GP practices and met the inclusion criteria were invited to participate. Including both invitees and their relatives, 20,198 volunteered and completed all aspects of the extensive phenotyping, which included pre-clinic questionnaires and a two-hour face-to-face assessment. Compared with the Scottish population, the sample had a higher proportion of females (59%) with an older mean age (49 years), better health, higher level of educational attainment, and less deprived socioeconomic status [23]. Sample comparison to the Scottish population is shown in Table 1 and has been further described previously [22, 23]. The participants’ Scottish Index of Multiple Deprivation 2009 (SIMD) score was ascertained from the first part of their postcode. The SIMD is a validated area-based measure of comparative socioeconomic deprivation comprising seven aspects: current income; employment; health; education, skills and training; geographic access; crime; and housing [25].

**Table 1:** Sociodemographic comparison of GS:SFHS and the Scottish population.

Characteristic		GS:SFHSN = 20,198	Scottish Population ^a^N = 5.2M
Median Age (y)	Male	49	37
	Female	49	39
Female Gender (%)		59	52
White Ethnicity (%)		99	98
Employment (%)	Employed	61.8	58.0*
	Retired	14.0	12.9*
Qualifications	Degree	29.8	20
	None	7.7	33
Cohabiting (%)		64.7	62^b^

* People aged 16–74 years

^a^ Scottish Executive (2012)

^b^ Scottish Executive (2006)

Here, we report the specific details of the mental health assessments.

### Ethics statement

The Tayside Research Ethics Committee (reference 05/S1401/89) provided ethical approval for the study. Participants all gave written consent, after having an opportunity to discuss the project, and before any data or samples were collected. The details of their consent status are recorded in the study database. All consent forms and study protocols were approved by the Research Ethics Committee. GS:SFHS data is available to researchers on application to the Generation Scotland Access Committee (access@generationscotland.org). The managed access process ensures that approval is granted only to research which comes under the terms of participant consent and privacy.

### Clinical Assessment

The in-person clinical visit included physical measurements, biological sampling, psychiatric (DSM-IV), mood state/psychological distress, personality, and cognitive assessment. Trained researchers administered the screening questions of the Structured Clinical Interview for DSM-IV Non-Patient Version (SCID) [26] and, if the screen was positive, they administered the mood sections of the SCID. Section A and the parts of Section D designed to exclude depressive episodes better explained by bipolar disorder, a general medical condition, or substance abuse were administered. Additional SCID question items designed to ascertain age of onset, number of episodes, and current episode were also administered. Interviews were conducted blind to the diagnostic status of related individuals. Participants who fulfilled criteria for Bipolar I Disorder (n = 75) were excluded from having an MDD diagnosis and marked “NA” in further analyses, but their relatives’ information was retained. The SCID elicited the presence or absence of a current or historical episode of MDD (n = 2706), the age of onset (AOO), and number of episodes suffered up until the point of interview, which allowed MDD categorisation into single (n = 1364) and recurrent (n = 1342) cases. Finally, individuals fulfilling the criteria for a major depressive episode (MDE) within the last month were identified (total n = 526, 173 single MDD, 334 recurrent MDD, 19 bipolar cases) by the SCID interview and were considered ‘current MDD’ cases.

### Interviewer Training and Quality Control

All interviewers received group training in the administration of the SCID (from DJM), and on-going refresher sessions throughout the study. Senior research nurses and academic psychiatrists at each site received extra training and acted as local mentors. A local training video was created to supplement the official SCID videos and training manual. Digital audio recordings (N = 58) of sequential clinic sessions were reviewed by DJM and AGM (blind to database diagnosis). Inter-rater reliability for the presence or absence of a lifetime diagnosis of major depressive disorder was good (Kappa = 0.86, p < 0.001, 95%CI 0.7 to 1.0).

## Statistical Methods

In a family study with pedigrees of varying size and structure, heritability, as the proportion of additive genetic to phenotypic variance, can be calculated using generalised linear mixed models. Pedigree-based heritability estimates take advantage of the phenotypic variability among family members. An individual is said to be ‘informative’ to the model when they have a non-missing phenotype (either case or control). An individual's pedigree relationships are most informative to the model in estimating genetic trait variance when that individual has at least one relative who is affected because it helps to constrain the model’s trait variance estimates between 0 and infinity. The number of informative pedigree relationships for MDD and for AOO analyses pedigree models is reported in Table 2.

**Table 2:** Number of informative relationships for MDD and AOO pedigree analysis in GS:SFHS.

	MDD	AOO
Total Number of relationships	28,040	3,466
Mother-child relationships	14,441	926
Father-child relationships	13,648	768
Full sibling relationships	9,038	380
Sibling relationships identified by the Mother	9,126	386
Sibling relationships identified by the Father	9,056	380
Maternal grandmother-grandchild relationships	2,980	137
Maternal grandfather-grandchild relationships	2,913	133
Paternal grandfather-grandchild relationships	1,627	46
Paternal grandmother-grandchild relationships	1,642	46

### Correlations Between Relatives

We calculated phenotypic correlations between kinship dyads (full siblings, parent-child, grandparent-grandchild, aunt/uncle-niece/nephew, and first cousin-first cousin). We created random subsets ("jackknifing") of the data where each family contributed only one dyadic pair to the subset so that larger families would not contribute more to the estimate. From each random subset we calculated the phenotypic correlation between pairs and we repeated this procedure 500 times to generate a mean and 95% confidence intervals for each kinship correlation.

### Variance Component Analyses

We estimated heritability using models that take into account all relationships based upon the full pedigree structure, and allow for unbalanced designs so that not every family has to have the same set of relationships [20]. In these models, the expected additive genetic relationship between all pairs of individuals is calculated from the pedigree and entered into a pairwise matrix (called the numerator relationship matrix or A matrix). This matrix is then used to condition a random effect from which the additive genetic variance is estimated. We treated MDD status as a binary response variable (i.e. 0/1). In order to overcome the limitations of restricted maximum likelihood methods with regard to non-Gaussian, binary response data [27], we estimated variance components using Bayesian methods as implemented in the MCMCglmm package for R [28]. MCMCglmm uses Markov chain Monte Carlo (MCMC) techniques to generate samples from the posterior distribution of each model parameter and supports likelihood models for non-Gaussian response variables, such as MDD.

To estimate the heritability of depression in our sample, we used a univariate model: MDD status (0 = absent, 1 = present, NA = bipolar) was the dependent variable in the model using a logit link function. We estimated unadjusted heritability using a model with only age and sex as covariates to get a sense of an upper boundary on our heritability estimate and because fixed effects that are genetically correlated with the trait, such as anxiety in MDD, can downwardly bias heritability estimates [29]. We then calculated an adjusted heritability from a model using all the sociodemographic correlates, and calculated another estimate from a model with an additional random effect for each family group. The family ID random effect would include individuals that reported themselves to be part of the same family at the time of interview. This definition of family ID would therefore include some married-in relatives (spouses) as well as genetic relatives such as siblings, parents, and cousins. This is a broadly defined family environment effect. This model was fitted to the data in order to capture non-genetic sources of extended-family similarity. Models were run to achieve acceptable parameter space sampling after a ‘burn in’ period. Four instances of each model were run and we checked for satisfactory model convergence by visually comparing sampling distributions from each run overlapped and testing whether they were indistinguishable [30]. For final parameter estimates we combined all the chains for a model together. For the adjusted general heritability model, we also included known sociodemographic correlates of depression: income, education, occupation, the Scottish Index of Multiple Deprivation (SIMD), smoking status, alcohol use status, and cohabitation with a partner. As all of these sociodemographic measures were assessed at the same time as the SCID MDD status, the temporality of the MDD episode versus the sociodemographic correlate was not known. We report heritability on the liability (or latent) scale [31, 32]; that is, $h^{2}=V_{A}/\left(\sum \;V+\frac{\pi^{2}}{3}\right)$ where V_A_ is the additive genetic variance, ∑V is the sum of all variance components, and π^2^/3 is the distribution-specific variance. We use a liability scale estimate for unobserved characters such as disease traits (0/1) where only the presence or absence of illness is ascertained, because we assume that the genes underlying these traits, if complex and additive in nature, will contribute incremental risk, or liability, to the illness [33, 34]. Thus, what one inherits is liability towards the illness, not the disease itself. We report the strength of association between MDD and sociodemographic factors by exponentiating the fixed effect regression coefficients to generate odds ratios [35]. We summarized parameter estimates using posterior means and 95% credible intervals (CI) using the region of highest posterior density. These intervals may be interpreted as the range of values in which there is a 95% probability that the true estimate lies, given the data and the priors. We determined the statistical significance of fixed effects using empirical p-values (pMCMC), which is the proportions of iterations in the MCMC sample that were above (or below) zero.

To estimate differences in the heritability of MDD in females and males, we used a bivariate model for MDD status by sex, where each individual had a 0 or 1 depending on their status in the column for their sex and a missing observation in the column for the other sex. We calculated sex-specific heritabilities and cross-sex genetic and shared environment correlations.

To estimate the genetic and shared environment correlations between age-of-onset (AOO) of MDD in our sample, we classified participants by status and AOO into 'absent', 'earlier onset', and 'later onset' with age 40 as the cut-off between earlier and later AOO. We used age 40 as the cut-off on the basis of initial definitions of onset subtypes [36–38] and because it helped maximize the sample size of the two age of onset subgroups. We could not use a later age of onset, for example of 60 years old, because our sample size of MDD cases at that threshold was not sufficient to have a well-powered analysis (n AOO ≥ 60 = 24). This is owed partly to the fact that this sample was recruited primarily at middle age. To handle separation in the data, where individuals who were assessed at age < 40 could not express the 'later onset' phenotype, we restricted the AOO analysis to participants who were older than 40 when assessed (n = 13,153). We fit a categorical model with 'absent' as the baseline with additive genetic and shared extended family environment as random effects in the pedigree model. Finally, we stratified the sample based on disease course into ‘absent’, ‘single’, and ‘recurrent’ MDD phenotypes to estimate the shared genetic and environmental variance between single and recurrent courses of MDD.

For all three stratification models (sex, AOO, and illness course) we fitted stratified depression as a categorical dependent variable. For the sex-based stratification we fit an interaction of sex with genetic and family environment variances. For the AOO and disease course models we specified two latent traits, each of which expressed a propensity for one of the affected statuses (earlier/later and single/recurrent) versus the baseline category of unaffected. This yielded a covariance matrix (variances for the affected categories and covariance between them) for each random term. From the AOO and illness course categorical models, we calculated the marginal heritability of each affected category excluding the other affected category. For example, when calculating the heritability of recurrent depression, the marginal heritability would be the heritability of recurrent MDD in comparison to being unaffected excluding the possibility of being a single MDD case, and vice versa.

### Inference to the Scottish Population

To make inferences from the sample to the population of Scotland, we reweighted each participant using age and sex frequencies in Scotland and the SIMD (which is calculated in quintiles so should be represented equally). To make an initial inference of population prevalence, we assumed that the combination of these three factors would be acceptable proxies for population frequencies of the other variables. For each participant we entered in the Scottish population frequency of their age and sex then divided this frequency by the number of study participants in each age/sex combination. We did the same for the SIMD. We created a similar inverse weight for each family group in the sample so that each family group contributed equally to the estimate. We then multiplied the age/sex, SIMD, and family weights together to create an individual weighting for each participant, then scaled the individual weightings to sum to 1. For each fixed effect predictor we multiplied the fitted coefficient by the sum of the individual weightings in that category and then added them all together to estimate the population mean. Since estimates from retrospective assessments may be downwardly biased [39], we also estimated an upper bound for the sample prevalence based on comparisons of rates between cumulative and retrospective studies [40] using a model programmed in Stan [41] (S1 Supplementary Methods).

## Results

In total 4,539 individuals of the full (N = 20,198) sample screened positively for emotional or psychiatric difficulties of whom 2,726 met DSM-IV criteria for current and/or past MDD using the SCID. This corresponds to a sample prevalence of 13.5%. Reweighting the sample based on population frequencies of age, sex, and SIMD, this is equivalent to an estimated population prevalence of 12.2% (CI 11.4%–13.1%). The affected status of individuals who screened positive for the SCID interview but then subsequently refused to undergo the SCID (N = 507), were excluded from further analysis and from sample prevalence estimates. According to the SCID interview, 507 individuals, or 2.4% of the sample, were experiencing a major depressive episode and not bipolar cases at the time of interview, which is approximately 18% of all subjects with an MDD diagnosis.

**Fig 1:**
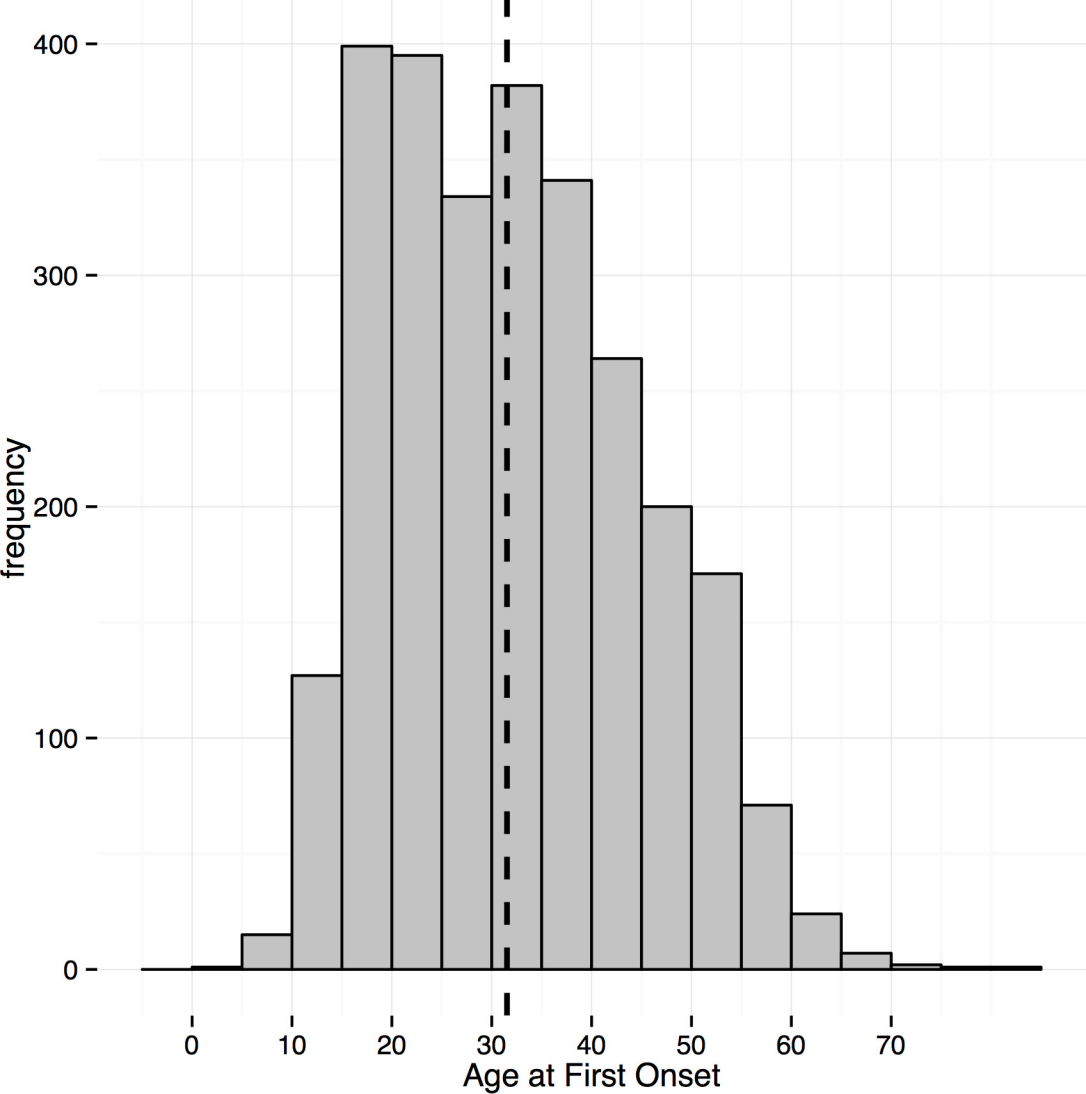
Age of onset distribution. Dashed line is the mean.

**Fig 2:**
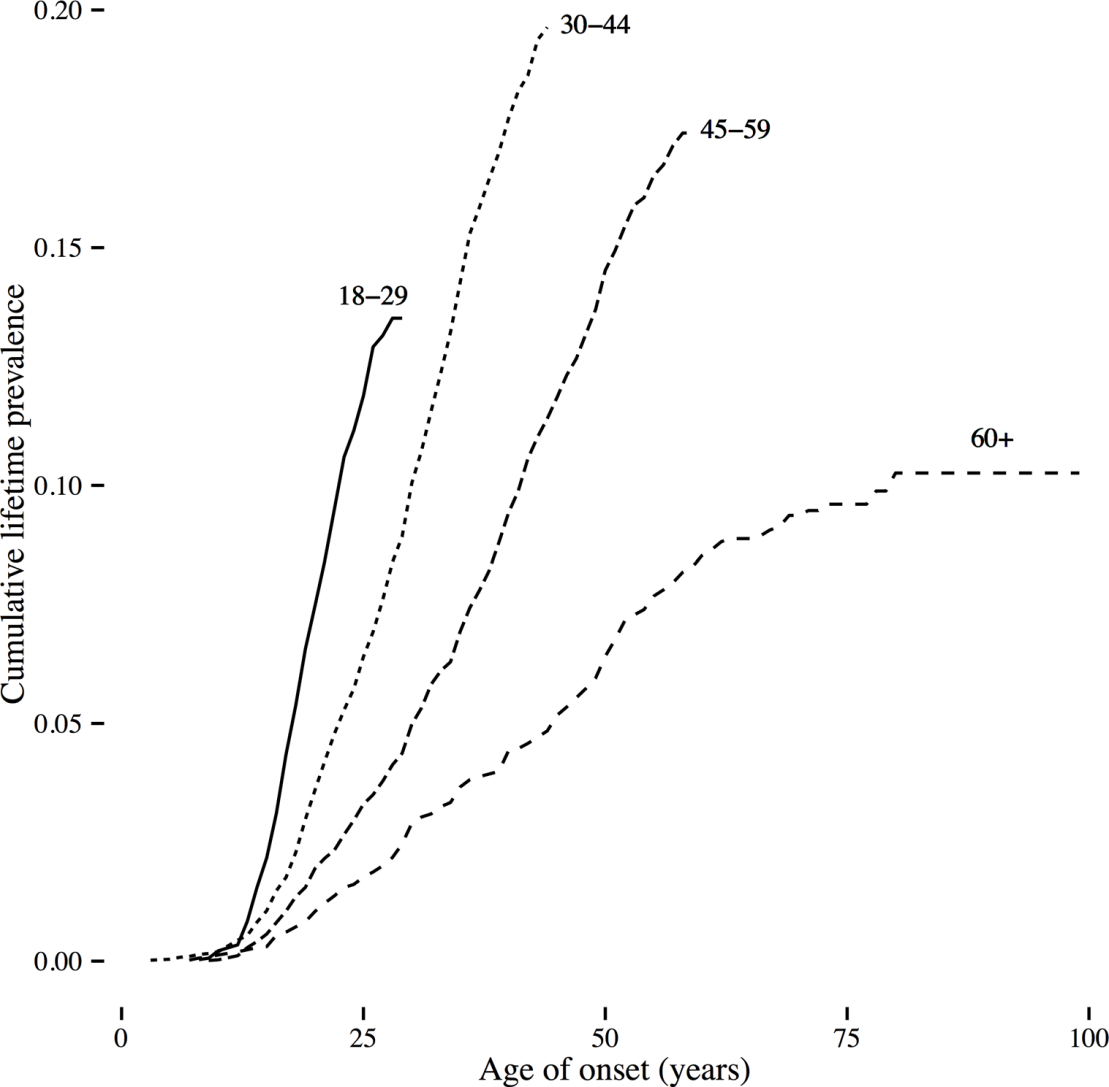
Kaplan-Meier survival curves for age of onset by age group.

The mean age of onset of MDD in GS was 31.7 years (SD 12.3, see Fig 1 for the distribution and S1 Table for the number of cases by age of onset). Thirty-five percent (35%) of SCID-diagnosed MDD cases had an age of onset of 25 or younger. Kaplan-Meier survival curves for age-of-onset of MDD were generated for 4 groups defined by age at interview (see Fig 2). The cumulative lifetime prevalence was highest in midlife for the age group at interview between 30–44 years of age. Overall risk increased from adolescence upwards in each age cohort. In order to assess whether age of onset was biased towards the age at interview, we graphed the regression from a generalized additive model fit to the reported age of onset data. We then compared this regression of the reported data to the uniform distribution of age of onset expected if onset was reported uniformly by participants after age 11 (See S1 Fig). The sample age of onset distribution shows some upward bias towards the age of interview of about 2–5 years compared to the expected uniform probability distribution, but the youngest interviewees do not show this bias. Taking into account the difference in prevalence between prospective and retrospective studies, we estimated that a prospective study design would yield a sample prevalence for depression of 33.0% (CI = 29.6%–36.5%).

### The heritability of MDD in GS:SFHS

**Fig 3:**
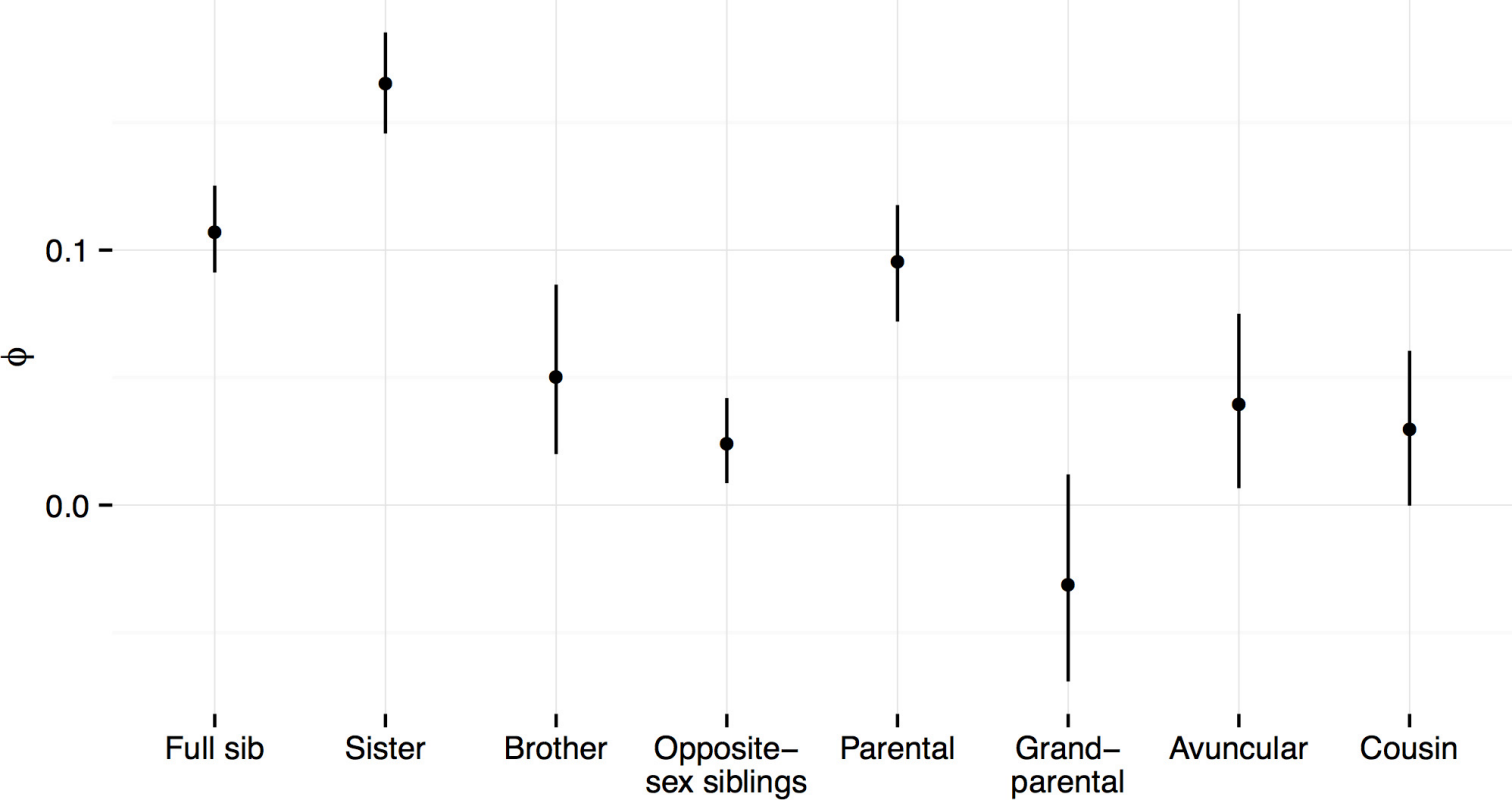
Phenotypic correlations of MDD status (absent = 0, affected = 1) between kinship dyads. Estimates from 500 jack-knifed replicates that sampled a single pair from each family for full siblings (N families with one or more pairs = 4306), full sisters (N = 2239), full brothers (N = 1161), opposite sex full-siblings (N = 2426), parent-child (N = 3402), grandparent-grandchild (N = 391), avuncular (aunt/uncle-niece/nephew N = 1826), first cousins (N = 1194).

The phenotypic correlations for each kinship dyad are plotted in Fig 3. The correlations ranged from *r* = 0.17 for full sisters to -0.03 for grandparent-grandchild pairs (S2 Table, Fig 3). The unadjusted heritability of MDD in GS:SFHS was 44% (0.44, CI: 0.37 to 0.52) on the liability scale, adjusted only for age and sex (Table 3). Heritability of MDD, after additional adjustment for sociodemographic factors (Table 3) was only slightly attenuated at 41% (0.41, CI: 0.32 to 0.50). When adjusting for the effects of shared family environment, which accounted for 7% (0.07, CI: 0.01 to 0.15) of the phenotypic variance in liability to MDD, the estimated heritability was reduced to 28% (0.28, CI: 0.12 to 0.47). Together the genetic and family environment effects accounted for 35% (0.35, CI = 0.24 to 0.48) of the phenotypic variance in liability to depression.

**Table 3:** Heritability, variance proportions, and stratified correlations of MDD. h^2^ = heritability, r_G_ = genetic correlation, c^2^ = shared family environment, r_C_ = shared family environment correlation.

Model	Trait	h^2^	r_G_	c^2^	r_C_
Unadjusted	MDD	0.44 (0.37, 0.52)	—-	—-	—-
Adjusted for Shared Family Environment	MDD	0.28 (0.12, 0.47)	—-	0.07 (0.01, 0.15)	—-
Adjusted for Sociodemographic Factors	MDD	0.41 (0.32, 0.50)	—-	—-	—-
Sex	Female MDD	0.44 (0.25, 0.61)	0.75 (0.43, 0.99)	0.04 (0.00, 0.13)	0.52 (-0.70, 1.00)
	Male MDD	0.35 (0.08, 0.63)		0.08 (0.00, 0.20)	
Age-of-onset	Earlier onset	0.21 (0.12, 0.28)	0.85 (0.66, 0.98)	0.04 (0.01, 0.07)	0.45 (-0.13, .88)
	Later onset	0.23 (0.13, 0.32)		0.04 (.01, .07)	
Course	Single episode	0.28 (0.14, 0.41)	0.86 (0.68, 0.97)	0.03 (0.00, 0.08)	0.50 (-0.48, 0.93)
	Recurrent episode	0.41 (0.20, 0.60)		0.07 (0.00, 0.16)	

The sex-specific heritability was 44% (0.44, CI: 0.25 to 0.61) for females and 35% (0.35, CI: 0.08 to 0.63) for males (Table 3), but was not significantly different (p = 0.58). The marginal heritabilities of earlier and later AOO were similar to the heritability when MDD was coded as absent/present and were not significantly different from each other (p = 0.93). The marginal heritabilities of single and recurrent episodes, in contrast, differed from each other. The marginal heritability of recurrent episode (0.41, CI: 0.20 to 0.60) was significantly higher (p < 0.0005) than that of single episode (0.28, CI: 0.14 to 0.41).

There was a strong, positive genetic correlation between MDD in males and females (0.75, CI: 0.43 to 0.99). There was also a strong, positive genetic correlation between earlier and later onset (0.85, CI: 0.66 to 0.98). Single episode and recurrent episode depression were also very strongly genetically correlated at 0.86 (CI: 0.68 to 0.97). These strong positive genetic correlations indicate that the genetic contribution to MDD is largely shared amongst these groups.

### Sociodemographic factors

Effects sizes for the sociodemographic factors are given in Table 4. The population prevalence of MDD in women was 15.8% (CI = 14.7% to 16.8%) and in men was 9.1% (CI = 8.3% to 9.9%); thus the attributable risk for women was a 6.7% (CI = 5.8% to 7.7%). Compared with being employed, being unemployed due to disability translated into an increase of 17.8% (CI = 13.1% to 22.8%) in the incidence of MDD, while being retired was a protective factor (attributable risk reduction of -4.0%, CI = -6.1% to—1.8%). Being a former drinker also conferred an increased risk for MDD (attributable risk 12.3%, CI = 9.5% to 15.2%).

**Table 4:** The effects of social and demographic variables on MDD risk.

Fixed Effects	Odds Ratio	95% Credible Interval	β	pMCMC
Sex: Male	1.00	-	-	-
Sex: Female	2.66	2.27–3.13	0.98***	<2e-04
Age	1.00	0.99–1.01	0.00	0.91
**INCOME**				
Between 10k-30k	1.00	-	-	-
Less than 10k	1.28	0.96–1.66	0.24	0.09
Between 30k-50k	0.78	0.64–0.94	-0.25*	0.01
Between 50k-70k	0.69	0.54–0.85	-0.38***	0.0008
More than 70k	0.64	0.47–0.81	-0.45***	0.0008
No response to income question	0.56	0.44–0.69	-0.58***	<2e-04
**EDUCATION**				
University Degree	1.00	-	-	-
Post-Secondary	1.03	0.85–1.22	0.03	0.76
Upper Secondary	0.68	0.53–0.86	-0.39**	0.001
Lower Secondary	0.74	0.57–0.93	-0.30*	0.01
Primary	0.65	0.48–0.83	-0.43**	0.002
Unknown Education	0.84	0.62–1.08	-0.19	0.17
**OCCUPATION**				
Employed	1.00	-	-	-
Student	0.66	0.42–0.93	-0.44*	0.03
Homemaker	1.20	0.79–1.61	0.16	0.34
Retired	0.59	0.45–0.73	-0.54***	<2e-04
Unemployed but seeking work	1.41	0.86–2.06	0.32	0.15
Unemployed Disabled	4.74	3.20–6.56	1.54***	<2e-04
No response to employment question	1.42	0.74–2.14	0.32	0.21
**COHABITING AS A COUPLE**				
Yes	1.00	-	-	-
No	1.34	1.14–1.57	0.29***	<2e-04
No response	1.14	0.61–1.76	0.10	0.69
**SCOTTISH INDEX OF MULTIPLE DEPRIVATION**				
Most deprived quintile	1.17	0.88–1.46	0.14	0.25
2^nd^ Most deprived quintile	1.03	0.80–1.28	0.02	0.88
Median quintile	1.00	-	-	-
2^nd^ Least deprived quintile	0.79	0.62–0.97	-0.24*	0.03
Least deprived quintile	0.92	0.73–1.12	-0.09	0.40
Quintile information unavailable	0.95	0.70–1.25	-0.06	0.68
**SMOKING STATUS**				
Non-smoker	1.00	-	-	-
Ex-smoker	1.58	1.33–1.83	0.45***	<2e-04
Current Smoker	2.82	2.29–3.41	1.03***	<2e-04
No response to smoking question	1.32	0.47–2.31	0.21	0.58
**ALCOHOL DRINKING STATUS**				
Non-drinker	1.00	-	-	-
Ex-drinker	4.93	2.70–7.69	1.56***	<2e-04
Current drinker	2.02	1.13–2.99	0.68**	0.004
No response to drinking question	2.19	0.96–3.77	0.72*	0.03

## Discussion

The estimated prevalence of lifetime MDD shows geographical variability, however recent studies in continental Europe, the USA, and Canada, suggest a range between 8.2% and 16.9% [2, 5, 42]. In the current study, we estimated the prevalence of MDD in Scotland to be 12.2%, consistent with this range of previous estimates. Prevalences in our sample were highest in midlife, consistent with previous studies [5]. The increased prevalence for MDD in early adulthood has been identified in previous studies and could be a result of both recall bias in older cohorts and increased prevalence in younger cohorts [43, 44] and these two factors are confounded in cross-sectional surveys [45] and thus our estimates are likely to be downwardly biased. While the MDD mean age of onset of 31.7 in our sample is consistent with another large epidemiological survey that used retrospective assessment of MDD which reported 32 years [46], the mean age of onset reflects the sample’s initial recruitment criteria at midlife. A previous study comparing retrospective recall with health service data indicated a very high correlation between the two methods of assessment for age of onset at 0.93 [47], indicating that this may be among the most reliable of MDD measurements, in the context of contact with professional mental health services and hospitalisation. Since lifetime prevalence estimates are known to be downwardly biased in retrospective studies [39, 40], with greater bias for episodic than for chronic disorders [40], our sample estimates of prevalence and age of onset likely reflect some bias that over represents current cases [40] and is confounded with the age recruitment and age distribution of the sample population [39, 48–50]. It is difficult to interpret the nature of bias with reporting the age of onset by the participants in our sample without longitudinal measures because it is not known whether or not the nature of episodic MDD should display a uniform distribution in age of onset. Assuming that the same factors influencing MDD recall in our retrospective study are the same as in other studies, we estimated that the lifetime prevalence of cumulatively identified MDD cases could be closer to 33% (CI = 29.6%–36.5%; see S1 Supplementary Methods). The level of confounding with age at interview and retrospective recall can be assessed in the future as the sample undergoes further waves of reassessment.

We found that the heritability of MDD in GS:SFHS, after accounting for shared family environment, was 28%. This estimate is outside the confidence intervals of the MDD heritability estimate of 37% from a meta-analysis of twin studies, where shared common environment can be difficult to account for [8]. This is expected as heritability estimates from pedigree samples are generally lower than those from twin samples [51]. Having a larger number of individuals per family from a pedigree also gives more power to detect shared family environment effects [52] compared with twin studies where only two individuals per family are generally observed. In a twin study where family environment effects are present but are not statistically different from zero and are subsequently dropped from the model, the heritability will be upwardly biased. The total variance explained by genetic and family environment effects of 35% was consistent with published twin heritability estimates [8]. Since the upper limit of explained trait variance is reduced by measurement error, the high unadjusted point estimate of heritability in GS:SFHS may reflect the robust phenotyping and quality control procedures established in this cohort.

In order to identify heterogeneity in MDD, we sought to stratify our sample using three criteria: sex, age of onset, and illness course. We found that earlier and later ages of onset had similar heritabilities, that were not significantly different, and were highly genetically correlated. Male and female MDD had non-significantly differing heritabilities that were also strongly positively correlated with one another, although the credibility intervals never overlapped with 1. Finally, single and recurrent illness course were also strongly genetically correlated with each other. Thus, age of onset, illness course, and sex probably do not represent genetically distinct subgroups of illness, although the credibility intervals of the estimates remain wide. However, intriguingly, the heritability of recurrent MDD was significantly larger than that for single MDD. This could be interpreted in two ways: while the same genetic variation is shared by both illness courses, there is a stronger genetic influence on MDD with a recurrent course. Alternatively, taking into consideration that while the genetic correlation is very high, it is still not unity. This could mean that recurrent MDD represents a clearer diagnosis of a more homogeneous disease. Single MDD may include some individuals who are not ill or have a different disorder, which results in the lower heritability and genetic correlation between single and recurrent of less than one. Single MDD may also include some individuals which have not yet experienced a second episode of MDD and are therefore ‘misclassified’ recurrent MDD cases. This kind of measurement error will also add noise to the model and decrease our ability to appropriately identify recurrent cases and limit our ability to accurately estimate the genetic correlation between single and recurrent MDD. Furthermore, separating components of variance and estimating effects in these models is difficult for binary traits as the modelling procedure requires us to fix the environmental variance in order to estimate the genetic variance. Repeated measures would go a long way to improve modelling because it would allow dissecting the effects of unique environment from measurement error.

While the estimated genetic correlation between depression in males and females was lower than for the other comparisons, the credibility intervals were wide for sexes and wider for males than for females. The genetic correlation of 0.75 still reflects a substantial degree of genetic overlap and the estimated heritability difference was non-significant. The higher heritability estimate for females is consistent with other work in the field [16], but the non-significance of the difference may be suggestive of other influences on this estimate such as prevalence and sex-specific environmental effects. The wider confidence intervals on the estimate of male MDD heritability in our sample may also be partly due to greater female participation in the overall study. The genetic correlation between males and females, combined with non-significant differences in heritability estimates, higher prevalence in women, and the higher phenotypic correlation between full sisters than full brothers suggests a sex-specific genetic aetiology [8, 15]. Future analyses will include models in which sex interactions are modelled together with illness course, AOO, family environment, and sociodemographic factors to explore whether these factors differ by sex in increasing risk.

Unmeasured environmental factors shared by extended families made a small but significant contribution to the liability in MDD in our sample (~7%). There is sometimes a worry with pedigree models that the family environmental effect modelled will be contaminated with variance that is rightfully part of the genetic effect since the two could be confounded. However, a strength of the family ID that we used to model common environment is that it included a large range of genetic relationships and also some non-genetic relationships (e.g. spouses and in-laws). This reduces that possibility that the family effect will draw variance from the genetic effect as the pedigree can better account for that proportion of the variance that is genetic with its finer-grain mapping of the genetic relationships than a family ID factor can account for. Thus, in this sample the family effect modelled is more likely to reflect the contribution of shared environment alone. We note that twin models frequently find small to negligible shared environment effects. This difference in our finding and twin models may be coming from the increased numbers of individuals measured per family in our sample with differing genetic relationships (parent-offspring, sibling, avuncular, etc). Twin models are (typically) only able to sample two individuals in a family who also have the same genetic relationship inside the family (full sibling or identical pair), which will decrease discrimination for the components of the model. This finding of a significant effect of shared environmental factor is unusual in a complex trait [53, 54] and highlights a possibly important aetiological role that common environments may have in MDD. This finding is consistent with the small effect of environments shared by siblings estimated from twin samples [8].

Our sociodemographic correlates of MDD are broadly consistent with previous studies in the UK [4], USA [5] and internationally [2, 55]. An unanticipated finding of our sample was the increased odds ratio of MDD among those unemployed due to disability. However, considering that, in the UK, suffering from MDD is grounds for declaring disability and receiving unemployment benefits due to this disability [56], this could be largely confounded with MDD in our Scottish sample. The finding of an association between MDD and former drinkers has also been observed [6], although past drinkers of alcohol may be subject to withdrawal phenomena which may mimic MDD. In the present study, the alcohol use question was a categorical response, not a continuous units consumption variable, so there could be additional variance masked by the noise in the broad category of ‘current drinkers’ which would include individuals who potentially classify as alcohol misusers as well as individuals who have only had an alcoholic drink a few times in their lives and the entire range in between. Still, the association between MDD and smoking is consistent with other studies [57, 58]. In this first wave of data collection of the GS:SFHS the individuals were only asked about the presence of these sociodemographic factors and the presence or absence of MDD up until the date of interview and the temporal order of these factors with regards to when the MDD episodes occurred was not ascertained. Longitudinal data is needed in order to investigate the temporality of these findings, establish the direction of causality, and address other methodological issues–such as confounding.

Most previous estimates of heritability of MDD have largely been based on twin samples [8], although there are increasing numbers of population samples available for calculating this statistic [12, 13]. While more recently available methods employ genotyping to calculate common SNP heritability in unrelated individuals [59, 60], these methods indicate that common SNPs do not explain all of the heritability twin studies have found and a large proportion of “missing heritability” remains unexplained. Family and twin estimates suggest that there is a component of heritability that segregates within families not associated with common SNPs: whether that may be explained by rare variants, epistasis, environmental transmission, gene by environment interactions, or some other factor remains to be determined.

Our ability to estimate the heritability of MDD using the pedigrees, while simultaneously modelling known environmental correlates, is a particular strength of GS:SFHS. Further investigation of familial aggregation of MDD may clarify some of the sources of familial contribution to the variance of MDD expression in a population. Family-based recruitment methods may bias heritability estimates upwards, especially if having a relative with MDD is more likely to result in a clinical referral or if comparison subjects are screened for any psychopathology. The current investigation avoided these difficulties, whilst controlling for age over a relatively short 8-year period. This also helped to reduce any potential age cohort effects [61]. Nevertheless, overall heritability of MDD was broadly comparable to other studies in which MDD has been ascertained using a structured clinical interview with a trained interviewer [62, 63]; McGuffin *et al*. 1996; Glahn *et al*. 2012) and in line with meta-analytic estimates [8].

In summary, MDD in the GS:SFHS is substantially heritable and shows similar risk associations with employment, marital status, alcohol and other variables previously reported in independent studies. These heritabilities were not substantially reduced by accounting for measured covariates, however shared family environment did reduce the estimated heritability of MDD. Subdivision of MDD did not clearly identify distinct genetic subgroups. While single and recurrent MDD course had a strongly positive genetic correlation, recurrent MDD course had a significantly larger genetic variance than single MDD course, which could be an amplified effect of the genetic component on recurrent MDD. These findings help to establish GS:SFHS as a valuable study for genetic linkage and association studies and point to future directions for effective stratification and phenotype refinement.

## Supporting Information

S1 Fig Age at SCID Interview vs. Reported Age of Onset (AOO). (DOCX)

S1 Supplementary Methods Cumulative vs. Retrospective Prevalence of Depression. (PDF)

S1 Table Number of MDD Cases by Age of Onset in GS:SFHS (DOCX)

S2 Table Jackknifed Phenotypic Correlations between MDD Status of Kinship Dyads. (DOCX)
