# Supplementary material for: Epidemiology and Heritability of Major Depressive Disorder, Stratified by Age of Onset, Sex, and Illness Course in Generation Scotland: Scottish Family Health Study (GS:SFHS)
Source: PLoS One. 2015 Nov 16;10(11):e0142197. doi: 10.1371/journal.pone.0142197 (PMC4646689; doi:10.1371/journal.pone.0142197)

**S1 Figure 1. Age at SCID interview versus reported age of onset (AOO).** Size of each point is scaled to the number of participants at each interview and onset age combination. Blue line is a regression from a generalized additive model fit to the data. Black line represents a basic comparison where the transition to depression process starts at age 11 (the 1% quintile of observed AOOs) and the probability of depression onset is the same at every age (AOO = 11/2 + 0.5 * Age at interview). Under the basic model the expected age of onset would be [Age at interview - 11]/2. For individuals who were younger when interviewed, the average observed AOO is very close to this basic model. At older ages at interview, the average reported age of onset is only slightly higher than this (2-5 years).
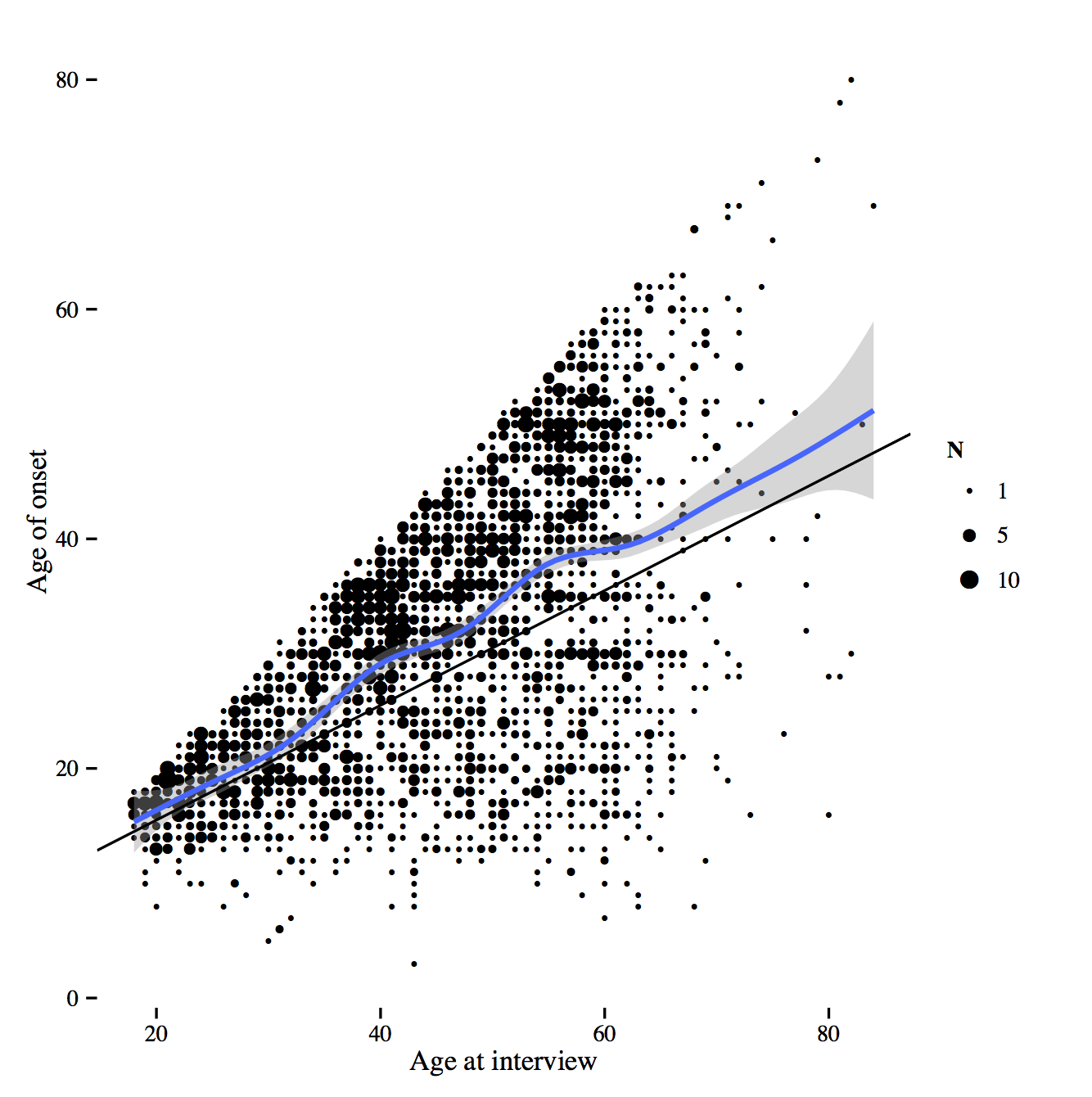

Supplement: S1 Fig — (DOCX) [file pone.0142197.s001.docx]
