# Supplementary material for: Epidemiology and Heritability of Major Depressive Disorder, Stratified by Age of Onset, Sex, and Illness Course in Generation Scotland: Scottish Family Health Study (GS:SFHS)
Source: PLoS One. 2015 Nov 16;10(11):e0142197. doi: 10.1371/journal.pone.0142197 (PMC4646689; doi:10.1371/journal.pone.0142197)
Supplement: S1 Supplementary Methods — (PDF) [file pone.0142197.s002.pdf]

Supplementary methods: Epidemiology and Heritability of Major Depressive Disorder, stratified by age of onset, sex, and illness course in Generation Scotland: Scottish Family Health Study (GS:SFHS)

AM Fernandes-Pujals et al

Cumulative vs retrospective prevalence of depression

We compared depression prevalence between studies that used cumulative or retrospective measures of depression to estimate the potential for the retrospective study design of Generation Scotland to yield a downwardly biased estimate of the prevalence.

```
set.seed(9181866) # from random.org
```

Data from:

Moffitt, T. E., Caspi, A., Taylor, A., Kokaua, J., Milne, B. J., Polanczyk, G., & Poulton, R. (2009). How common are common mental disorders? Evidence that lifetime prevalence rates are doubled by prospective versus retrospective ascertainment. Psychological Medicine, 40(06), 899–909.

Data from Table 2: depression

```
dunedin_ci = c(38.3, 44.5)/100
dunedin_mean = 41.4/100 # prevalence
dunedin_cases = 414 # number of cases

nzmhs_ci = c(16.7, 20.2)/100
nzmhs_mean = 18.5/100
nzmhs_cases = 621
```

```
ncsr_ci = c(17.5, 20.6)/100
ncsr_mean = 19/100
ncsr_cases = 526

ncs_ci = c(14.9, 19)/100
ncs_mean = 16.9/100
ncs_cases = 582
```

Data from Generation Scotland

```
gs_cases = 2726
gs_n = 20198
```

For each study we can use the reported prevalence and number of cases to parameterize a beta distribution describing the result

```
# beta distribution parameters based on data and uniform prior
beta_posterior <- function(prevalence, n_cases, a=1, b=1) {

  N <- n_cases / prevalence

  a = n_cases + a
  b = N - n_cases + b

  return(list(a=a, b=b))

}
```

calculate beta distributions

```
dunedin_beta <- beta_posterior(dunedin_mean, dunedin_cases)
dunedin_beta
```

```
## $a
## [1] 415
##
## $b
## [1] 587
```

```
nzmhs_beta <- beta_posterior(nzmhs_mean, nzmhs_cases)
nzmhs_beta
```

```
## $a
## [1] 622
##
## $b
## [1] 2736.757

ncsr_beta <- beta_posterior(ncsr_mean, ncsr_cases)
ncsr_beta

## $a
## [1] 527
##
## $b
## [1] 2243.421

ncs_beta <- beta_posterior(ncs_mean, ncs_cases)
ncs_beta

## $a
## [1] 583
##
## $b
## [1] 2862.787

gs_beta <- beta_posterior(gs_cases/gs_n, gs_cases)
gs_beta

## $a
## [1] 2727
##
## $b
## [1] 17473
```

Plot the retrospective and prospective prevalences.

```
xx <- seq(0.10, 0.5, by=.001)
plot(xx, dbeta(xx, shape1=dunedin_beta$a, shape2=dunedin_beta$b), type='l',
     main='Prevalence', ylim=c(0, 175), xlab='Prev', ylab='density', col="black")
lines(xx, dbeta(xx, shape1=nzmhs_beta$a, shape2=nzmhs_beta$b), col='#a6cee3')
lines(xx, dbeta(xx, shape1=ncsr_beta$a, shape2=ncsr_beta$b), col='#b2df8a')
lines(xx, dbeta(xx, shape1=ncs_beta$a, shape2=ncs_beta$b), col='#33a02c')
lines(xx, dbeta(xx, shape1=gs_beta$a, shape2=gs_beta$b), col='#fb9a99')

legend('topright', c('Dunedin', 'NZMHS', 'NCS-R', 'NCS', 'GS'),
                   col=c('black','#a6cee3',  '#b2df8a', '#33a02c', '#fb9a99'), lty=1)
```

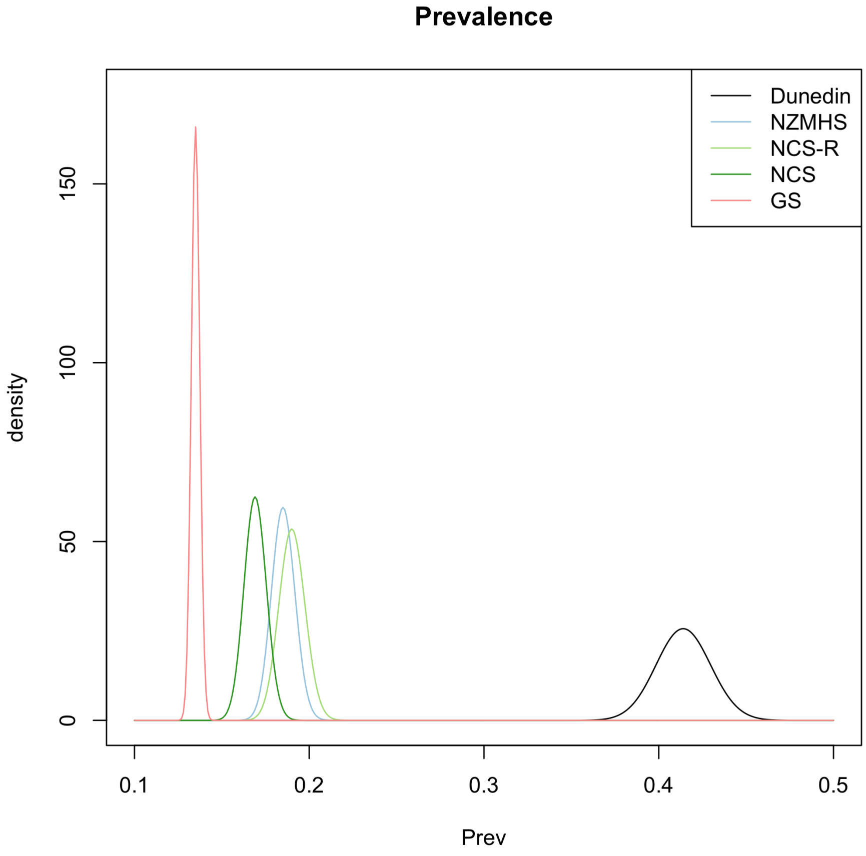

Figure 1: Uncertainty intervals for sample prevalence of MDD. (GS = Generation Scotland).

Logit model

We fitted a binomial model where the number of cases in the retrospective studies for study i was $\text{cases}_{ij} \sim \text{BinomLogit}(N, \mu + u_{j[i]} + b \times \text{cumulative}_i)$ where cumulative_i was 1 if the study is the cumulative Dunedin study and 0 otherwise; and u were the country intercepts with $u_{j[i]}$ the j th country intercept (1 = New Zealand [Dunedin and NZMHS], 2 = USA [NCS and NCS-R], and 3 = Scotland [Generation Scotland]). The parameter of interest is b, the slope parameter for the effect of a cumulative study design.

Get data into a single data frame

```
moffitt <- data.frame(study=c('Dunedin', 'NZMHS', 'NCS-R', 'NCS'),
        prevalence=c(dunedin_mean, nzmhs_mean, ncsr_mean, ncs_mean),
        cases=c(dunedin_cases, nzmhs_cases, ncsr_cases, ncs_cases),
        cumulative=c(1, 0, 0, 0),
        country=c(1, 1, 2, 2))

# determine total sample size in each study
moffitt <- transform(moffitt, N=round(cases / prevalence))

genscot <- data.frame(study='GS', prevalence=0.135,
                      cases=2726, cumulative=0,
                      country=3, N=20198)

mdd <- rbind(moffitt, genscot)
mdd
```

```
##     study prevalence cases cumulative country     N
## 1 Dunedin      0.414   414          1       1  1000
## 2   NZMHS      0.185   621          0       1  3357
## 3   NCS-R      0.190   526          0       2  2768
## 4     NCS      0.169   582          0       2  3444
## 5      GS      0.135  2726          0       3 20198
```

Determine a reasonable SD for the country intercepts so that the retrospective studies are captured but the cumulative study is clearly outside it, so that the constant intercept has to be close to the average of the retrospective studies and is not pulled up by the cumulative estimate.

```
# transform to logit scale
l0 <- arm::logit(mdd$prevalence)

# centre on the average of the retrospective studies
u0 <- l0 - mean(l0[2:5])
```

```
sd(u0[2:5])
```

```
## [1] 0.1848323
```

```
pnorm(u0, sd=0.2)
```

```
## [1] 1.00000000 0.71383520 0.76691795 0.50603574 0.09535387
```

```
pnorm(scale(l0, scale=FALSE), sd=0.2)
```

```
##              [,1]
## [1,] 0.999999703
## [2,] 0.247088832
## [3,] 0.301682078
## [4,] 0.108755555
## [5,] 0.005282125
## attr(,"scaled:center")
## [1] -1.346096
```

We will parameterize the country varying-intercepts with an SD of 0.2.

We implemented the model in the probabilistic programming language Stan.

```
library(rstan)
rstan_options(auto_write = TRUE)
options(mc.cores = parallel::detectCores())
```

The program is made up of four parts:

- a data statement with the number of cases and sample size of each study, a predictor for whether they study was cumulative or retrospective, and an index for which country the study was conducted in.
- a parameter statement for the mean prevalence (fixed intercept on the latent scale), three country-level (varying) intercepts, and a fixed slope for the cumulative study design effect.
- A model statement describing the relationship between the parameters and the data: $\text{cases}_{ij} \sim \text{BinomLogit}(N, \mu + u_{j[i]} + b * \text{cumulative}_i)$ and $u_j \sim \text{Normal}(0, 0.2)$. By default $\mu \sim \text{Uniform}(-\infty, +\infty)$ and $b \sim \text{Uniform}(-\infty, +\infty)$
- A replicate data statement for the estimate of the cumulative and retrospective sample prevalences for each of the three countries that the studies were conducted in, $\text{Retrospective}_j = \text{logit}^{-i}(\mu + u_j)$ and $\text{Cumulative}_j = \text{logit}^{-i}(\mu + u_j + b)$.

The program then uses MCMC to generate samples from the posterior distribution of each parameter.

```
prev_model <-
'
data {
        int<lower=0> cases[5]; // number of cases
        int<lower=0> N[5]; // total sample
        real<lower=0,upper=1> cumulative[5]; // 0 = retrospective, 1 = cumulative
        int<lower=0> country[5]; // index for country intercepts
}
parameters {
        real mu; // retrospective prevalence (mean)
        real u0[3]; // country intercepts (NZ, USA, Scotland)
        real b; // factor for cumulative prevalence
}
model {
        u0 ~ normal(0, 0.2);
        for(i in 1:5) {
          cases[i] ~ binomial_logit(N[i], mu + u0[country[i]] + b * cumulative[i]);
        }
}
generated quantities {
        // model-implied retrospective and cumulative prevalences
        real retro_rep[3];
        real cumul_rep[3];
        for(j in 1:3) {
          retro_rep[j] <- inv_logit(mu + u0[j]);
          cumul_rep[j] <- inv_logit(mu + u0[j] + b);
        }
}
'
```

```
prev_dat <- as.list(mdd[,c('cases', 'N', 'cumulative', 'country')])

prev_fit <- stan(model_name='prevalence (varying intercepts)',
                 model_code=prev_model, data=prev_dat,
                 iter=4000, chains=4, verbose=FALSE)
```

```
## COMPILING THE C++ CODE FOR MODEL 'prevalence (varying intercepts)' NOW.
```

```
print(prev_fit)
```

```
## Inference for Stan model: prevalence (varying intercepts).
```

```
## 4 chains, each with iter=4000; warmup=2000; thin=1;
## post-warmup draws per chain=2000, total post-warmup draws=8000.
##
##                    mean se_mean   sd      2.5%       25%       50%
## mu                 -1.63    0.00 0.12     -1.86     -1.71     -1.62
## u0[1]               0.14    0.00 0.12     -0.10      0.05      0.14
## u0[2]               0.10    0.00 0.12     -0.13      0.01      0.10
## u0[3]              -0.23    0.00 0.12     -0.46     -0.31     -0.23
## b                   1.14    0.00 0.08      0.99      1.09      1.14
## retro_rep[1]        0.18    0.00 0.01      0.17      0.18      0.18
## retro_rep[2]        0.18    0.00 0.00      0.17      0.17      0.18
## retro_rep[3]        0.14    0.00 0.00      0.13      0.13      0.14
## cumul_rep[1]        0.41    0.00 0.02      0.38      0.40      0.41
## cumul_rep[2]        0.40    0.00 0.02      0.37      0.39      0.40
## cumul_rep[3]        0.33    0.00 0.02      0.29      0.32      0.33
## lp__           -13194.86    0.03 1.57 -13198.74 -13195.66 -13194.54
##                      75%     97.5% n_eff Rhat
## mu                 -1.54     -1.40  1204 1.00
## u0[1]               0.22      0.37  1280 1.00
## u0[2]               0.18      0.33  1190 1.01
## u0[3]              -0.15      0.00  1221 1.01
## b                   1.20      1.29  2520 1.00
## retro_rep[1]        0.19      0.20  3959 1.00
## retro_rep[2]        0.18      0.19  7231 1.00
## retro_rep[3]        0.14      0.14  8000 1.00
## cumul_rep[1]        0.42      0.44  3232 1.00
## cumul_rep[2]        0.42      0.44  2747 1.00
## cumul_rep[3]        0.34      0.37  2625 1.00
## lp__           -13193.70 -13192.80  2056 1.00
##
## Samples were drawn using NUTS(diag_e) at Mon Sep  7 09:30:23 2015.
## For each parameter, n_eff is a crude measure of effective sample size,
## and Rhat is the potential scale reduction factor on split chains (at
## convergence, Rhat=1).
```

```
plot(prev_fit, pars=c('retro_rep', 'cumul_rep'))
```

The inferred cumulative sample prevalence of MDD in the Generation Scotland sample can be read from the `cumul_rep[3]` parameter.

```
print(prev_fit, pars='cumul_rep[3]', probs=c(0.025, 0.975), digits_summary=3)
```

```
## Inference for Stan model: prevalence (varying intercepts).
## 4 chains, each with iter=4000; warmup=2000; thin=1;
## post-warmup draws per chain=2000, total post-warmup draws=8000.
```

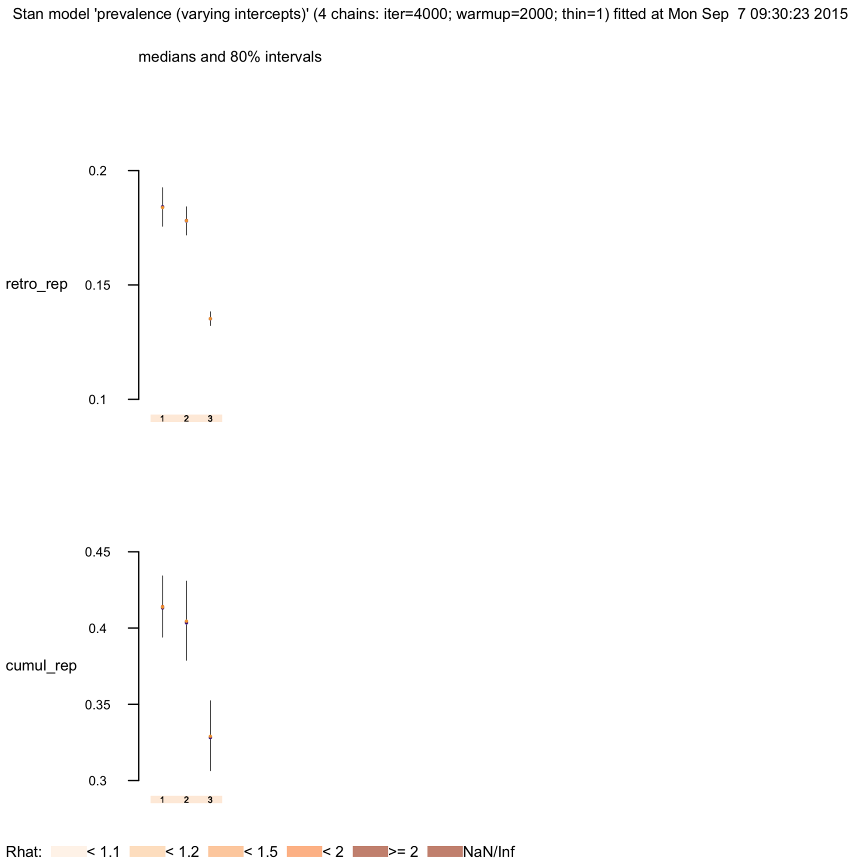

Figure 2: Parameter estimates for retrospective and cumulative MDD prevalences for New Zealand (1), USA (2) and Scotland (3)

```
##
##                mean se_mean    sd  2.5% 97.5% n_eff Rhat
## cumul_rep[3] 0.329       0 0.018 0.294 0.366  2625    1
##
## Samples were drawn using NUTS(diag_e) at Mon Sep  7 09:30:23 2015.
## For each parameter, n_eff is a crude measure of effective sample size,
## and Rhat is the potential scale reduction factor on split chains (at
## convergence, Rhat=1).
```
