# Supplementary material for: Epidemiology and Heritability of Major Depressive Disorder, Stratified by Age of Onset, Sex, and Illness Course in Generation Scotland: Scottish Family Health Study (GS:SFHS)
Source: PLoS One. 2015 Nov 16;10(11):e0142197. doi: 10.1371/journal.pone.0142197 (PMC4646689; doi:10.1371/journal.pone.0142197)
Supplement: S1 Table — (DOCX) [file pone.0142197.s003.docx]

| **S1 Table 1: Number of MDD Cases by Age of Onset in GS:SFHS** | | | | |
| --- | --- | --- | --- | --- |
| **AOO under age** | **n** |  | **AOO over age** | **n** |
| 15 | 177 |  | 15 | 2444 |
| 20 | 599 |  | 20 | 2022 |
| 25 | 954 |  | 25 | 1667 |
| 30 | 1321 |  | 30 | 1300 |
| 35 | 1670 |  | 35 | 951 |
| 40 | 1975 |  | 40 | 646 |
| 45 | 2199 |  | 45 | 422 |
| 50 | 2410 |  | 50 | 211 |
| 55 | 2543 |  | 55 | 78 |
| 60 | 2597 |  | 60 | 24 |
