# Supplementary material for: Epidemiology and Heritability of Major Depressive Disorder, Stratified by Age of Onset, Sex, and Illness Course in Generation Scotland: Scottish Family Health Study (GS:SFHS)
Source: PLoS One. 2015 Nov 16;10(11):e0142197. doi: 10.1371/journal.pone.0142197 (PMC4646689; doi:10.1371/journal.pone.0142197)
Supplement: S2 Table — (DOCX) [file pone.0142197.s004.docx]

**S2 Table 2:** Jackknifed phenotypic correlations between MDD status of kinship dyads.

| **Dyad** | ***r*** | **95% CI** | | **N** |
| --- | --- | --- | --- | --- |
| Full sibling | 0.11 | 0.09 | 0.13 | 4306 |
| Sisters | 0.17 | 0.15 | 0.19 | 2239 |
| Brothers | 0.05 | 0.02 | 0.09 | 1161 |
| Opposite-sex sibling | 0.02 | 0.01 | 0.04 | 2426 |
| Parental | 0.10 | 0.07 | 0.12 | 3402 |
| Grandparental | -0.03 | -0.07 | 0.01 | 391 |
| Avuncular | 0.04 | 0.01 | 0.08 | 1826 |
| Cousin | 0.03 | 0.00 | 0.06 | 1194 |

N = total number of families with at least one pair from the dyad.
